# Application of nano-radiosensitizers in non-small cell lung cancer

**DOI:** 10.3389/fonc.2024.1372780

**Published:** 2024-04-05

**Authors:** Xiao Hu, Jiamiao Hu, Yuke Pang, Mengjia Wang, Weiwen Zhou, Xuyun Xie, Chu Zhu, Xuanxuan Wang, Xiaonan Sun

**Affiliations:** Department of Radiation Oncology, Sir Run Run Shaw Hospital, Zhejiang University, School of Medicine, Hangzhou, China

**Keywords:** radiotherapy, nanomaterials, non-small cell lung cancer, radiosensitization, chemotherapy

## Abstract

Radiotherapy stands as a cornerstone in the treatment of numerous malignant tumors, including non-small cell lung cancer. However, the critical challenge of amplifying the tumoricidal effectiveness of radiotherapy while minimizing collateral damage to healthy tissues remains an area of significant research interest. Radiosensitizers, by methods such as amplifying DNA damage and fostering the creation of free radicals, play a pivotal role in enhancing the destructive impact of radiotherapy on tumors. Over recent decades, nano-dimensional radiosensitizers have emerged as a notable advancement. Their mechanisms include cell cycle arrest in the G2/M phase, combating tumor hypoxia, and others, thereby enhancing the efficacy of radiotherapy. This review delves into the evolving landscape of nanomaterials used for radiosensitization in non-small cell lung cancer. It provides insights into the current research progress and critically examines the challenges and future prospects within this burgeoning field.

## 1 Introduction

Radiotherapy, a prevalent treatment for non-small cell lung cancer (NSCLC) and various other malignant tumors, is often used in conjunction with chemotherapy and other treatments (1–3). Its fundamental principle involves the interaction of ionizing radiation with tumor cell components, either directly or indirectly. Direct interaction leads to the damage of critical biological molecules like DNA and proteins, hindering cell division and proliferation, ultimately causing cell death. Indirectly, radiation induces the production of reactive oxygen species (ROS) and free radicals, disrupting these biological molecules (4).

However, the use of radiotherapy encounters many challenges. Factors such as tumor stem cells, tumor heterogeneity, and angiogenesis can limit its effectiveness. Moreover, complications may arise, making it difficult for patients to tolerate prolonged radiotherapy (5). A strategic approach to surmount these challenges involves the use of radiosensitizers, which are designed to enhance radiotherapy’s efficacy while mitigating side effects on normal tissue (6).

In recent years, nanoparticles, known for their excellent biocompatibility, high drug loading capacity, and robust tumor permeability and retention (7, 8), have become a focal point in the realm of tumor radiosensitization. When delivered to tumors, these nanoparticles not only exert their therapeutic effects but also sensitize tumor cells to radiotherapy through various mechanisms. This review delves into the advancements in nano-radiosensitizers, particularly focusing on their underlying mechanisms and contributions to enhancing radiotherapy outcomes.

## 2 Nano-radiosensitizer

### 2.1 Metal nano-radiosensitizers

#### 2.1.1 Gold nanoparticles

Metal materials have been used in radiotherapy research for decades. In a pioneering study by Regulla in 1998, it was observed that mouse embryonic fibroblasts irradiated with X-rays on a gold surface exhibited increased biological effects compared to those in a tissue-like environment (9). Further research by Herold et al. revealed enhanced radiation effects in cancer cells with gold particles (10). By 2010, T. Marques et al. demonstrated that gold nanoparticles (GNPs) in tissues could selectively increase radiation doses to target areas, illustrating the potential of metals in refining radiotherapy (11).

Cuihong Wang et al. were the first to use thio-glucose-bound gold nanoparticles (Glu-GNPs) on NSCLC cells. They found that glucose enhances the uptake of Glu-GNPs by A549 cells, leading to their accumulation in vesicular endosomes or lysosomes. Upon X-ray exposure, Glu-GNPs triggered cell apoptosis through the modulation of Bcl-2 family proteins and activation of the mitochondrial apoptotic pathway (12). Tao Li’s team also worked with Glu-GNPs in A549 cells, achieving sensitization enhancement ratios (SER) of 1.41 and 1.15 for 160 kV and 6 MV X-rays, respectively (13). Shokouhozaman’s research indicated increased inhibitory effects of Glu-GNPs on QU-DB lung cancer cells by 64.4% and 32.4% under 100 kV and 6 MV X-rays (14). These findings proposed that Glu-GNPs may possess superior efficacy in combating NSCLC cells under conditions of low-energy radiation. However, the substantiation of these claims is somewhat feeble, primarily due to the omission of *in vivo* experiments by the researchers.

Additionally, GNPs have demonstrated potential in impeding the migratory capabilities of A549 cells post-radiation therapy, perhaps as a result of alterations in the cytoskeleton affecting overall cellular adhesion (15). Nevertheless, it would behoove the research to consider testing a broader range of dosages, extending beyond the used levels of 2Gy and 5Gy. Moreover, albumin-bound GNPs, known for their favorable biosafety profile, exhibited radiosensitization and anti-tumor activity both *in vivo* and *in vitro* experiments, boasting a SER of 1.432 (16). The study could be strengthened by incorporating more tumor models beyond A549 for validation. Zhongli Cai’s team found that the dose enhancement ratio of GNPs was lower in 3D culture models compared to single-monolayer (1.3 vs 1.6) (17). Sherif et al. argued that the common algorithm for calculating the dose enhancement ratio is overly simplistic and fails to consider specific profiles, leading to a decrease in the dose enhancement ratio due to potential rupture and detachment of GNPs’ surface coating (18). Therefore, optimizing the surface coating of GNPs was crucial, although it would certainly be better if the models were more closely aligned with the actual lung environment.

Fatma et al. modified GNPs with Schiff bases derived from galactose, resulting in larger particles that showed greater radiosensitization in A549 cells compared to unmodified GNPs (19). Arvind’s study compared 3.9 and 37.4 nm GNPs in Lewis lung cancer cells, finding significant radiosensitizing effects post-X-ray irradiation with both sizes, but no significant difference between them (20). Both studies might benefit from the inclusion of animal experiments and a broader spectrum of radiotherapy dose configurations.

**Table 1** summarizes researches on GNP combined with radiotherapy in NSCLC. These researches are extensive, indicating that particle size, coating, and surface modifiers significantly influence their radiosensitizing effect. Future studies are needed to optimize these factors for better clinical application.

**Table 1:** Types of GNPs used in combination with IR in NSCLC.

Nanoparticles	Cells	Mechanism	Outcome	Reference
Glu-GNPs	A549	Promotes apoptosis via Bcl-2 family proteins and mitochondrial pathway	Increased apoptosis under X-ray irradiation	(12)
Glu-GNPs	A549	Increased DNA double-strand breaks	SER of 1.41 and 1.15 for 160 kV and 6 MV X-rays	(13)
Glu-GNPs	QU-DB	Enhanced sensitivity to low-energy X-rays in NSCLC cells	Inhibitory effects increased by 64.4% (100 kV) and 32.4% (6 MV)	(14)
GNPs	A549	Affects cytoskeleton, alters cell adhesion, inhibits cell migration	Reduced migration ability post-radiotherapy	(15)
Albumin-bound GNPs	A549	Favorable biosafety profile, enhance radiotherapy	SER of 1.432 in A549 cells, exhibited radiosensitization and anti-tumor activity both *in vivo* and *in vitro* experiments	(16)
Schiff bases -modified GNPs	A549	Larger particle size, significant radiosensitization	More significant effect than unmodified GNPs	(19)
GNPs	Lewis	Increased DNA damage	significant radiosensitizing effects with both 3.9 and 37.4 nm GNPs	(20)

##### 2.1.1.1 Other metal nanoparticles

In the realm of NSCLC radiosensitization, silver nanoparticles (AgNPs) and gadolinium nanoparticles (AGuIX) have also shown promise. Gowda et al. discovered that AgNPs, when modified with gallic acid, effectively inhibited the expression of epithelial-mesenchymal transition markers induced by X-ray in A549 cells like Vimentin and N-cadherin, simultaneously promoting E-cadherin upregulation. This modification thereby reduced tumor cell radioresistance (21). Reetta’s team, in their study of AgNPs across multiple NSCLC cell lines, observed that these nanoparticles led to cell cycle arrest in different phases (A549 and Calu-1 cells in G2 phase; BEAS-2B cells in S phase) and increased ROS production and protein oxidation in cell mitochondria, thus elevating the cells’ sensitivity to radiotherapy. It’s noteworthy, however, that these nanoparticles did not modify the mitochondrial redox profiles altered by radiation therapy (22). Adding GNPs as a horizontal comparison would be more meaningful when studying AgNPs, as gold and silver are both precious metals.

AGuIX, a polysiloxane nanoparticle containing gadolinium ions (Gd3+), was first applied in H1299 and A549 cell. Applied initially to H1299 and A549 cells, Upon X-ray exposure, AGuIX produced photoelectrons, reactive oxygen species, and free radicals, leading to G2/M phase arrest and enhancing both radiosensitization and apoptosis in NSCLC cells (23). Wu Liu’s team innovated further by attaching gadolinium nanoparticles to a pH-low insertion peptide, boosting cellular Gd uptake dramatically and prolonging its tumor residence, significantly improving radiosensitivity in A549 cells (24). The design and validation of these two studies are fairly comprehensive, and we anticipate more profound research in the future.

Chaebin et al. developed gadolinium-embedded carbon dots (Gd@C-dots) via hydrothermal reaction, which compared to AGuIX, showed reduced toxicity due to lower Gd leakage *in vivo* and enhanced radiosensitivity in H1299 cells due to the catalytic properties of carbon (25). Another research compared CA or amino (pPD)-modified Gd@C-dots, with the pPD-modified Gd@C-dots demonstrating better uptake and retention in H1299 cells, indicating a higher potential for clinical application (26). Both studies are considerably thorough, however, there is room for further refinement particularly in the realms of in-situ tumor models or dose escalation studies.

CuPRiX, created by partially dehydrogenating Gd from DOTAGA(Gd) within AGuIX in an acidic environment, resulted in the unchelated Gd chelating free Cu in A549 cells. This led to the inhibition of the copper oxide-dependent enzyme (LOX) and reduced cell migration, thereby improving tumor radiosensitization over AGuIX (27). Research on copper nanoparticles is sparse, marking a novel aspect of this study. However, it is apparent that this research could benefit from a deeper exploration of the underlying mechanisms. Currently, AGuIX is undergoing phase I clinical trials for brain metastases and gliomas (28, 29), with research on its application in NSCLC still in the early stages. Below is **Table 2** concluding metal nanoparticles above.

**Table 2:** Types of other metal nanoparticles used in combination with IR in NSCLC.

Nanoparticles	Cells	Mechanism	Treatment Outcome	Reference
GA-modified AgNPs	A549	Inhibits epithelial-mesenchymal transition markers, promotes E-cadherin upregulation	Reduces tumor cell radioresistance	(21)
AgNPs	A549, Calu-1, BEAS-2B	Induces cell cycle arrest, increases ROS production and protein oxidation	Elevates sensitivity to radiotherapy	(22)
AGuIX	H1299, A549	Generates photoelectrons, ROS, and free radicals; triggers G2/M phase arrest	Enhances radiosensitization and apoptosis	(23)
pH-low peptide inserted Gd NPs	A549	Boosts cellular Gd uptake, prolongs tumor residence	Significantly enhances radiosensitivity	(24)
Gd@C-dots	H1299	Reduces toxicity, catalytic properties of carbon enhance radiosensitivity	Enhanced radiosensitivity, reduced Gd leakage	(25)
Gd@C-dots (pPD-modified)	H1299	Better uptake and retention	Indicates higher potential for clinical applications	(26)
CuPRiX	A549	Inhibits LOX enzyme, reduces cell migration	Improves tumor radiosensitization over AGuIX	(27)

### 2.2 Semiconductor nano-radiosensitizer

In the field of NSCLC radiosensitization, semiconductor nanoparticles have gained prominence. TiO_2_ nanoparticles, when excited by high Cerenkov radiation from X-rays, form electron-hole pairs that trigger the production of ROS, leading to DNA damage. Utilizing this principle, Zi Ouyang’s team designed TiO_2_ nanoparticles that significantly heightened the radiosensitivity of A549 cells (30). However, the lack of *in vivo* experiments was a shortcoming of this study.

Similarly, semiconductor zinc oxide (ZnO) exhibits comparable properties. Masoumeh wt al. developed ZnO nanoparticles, doping them with gadolinium to create Gd-ZnO-NPs. At concentrations of 10 and 20 μg/mL in SKLC-6 cells, these nanoparticles showed SER of 1.47 and 1.61, respectively, demonstrating a concentration-dependent increase in radiosensitivity. Flow cytometry analysis revealed that Gd-ZnO-NPs elevated apoptosis in NSCLC cells and caused more cells to arrest in G1 phase. Combined with X-rays, these nanoparticles downregulated the mRNA levels of DNA damage repair genes such as XRCC2 and XRCC4, hindering DNA repair and leading to increased cell death (31). Additionally, Gd-ZnO NP enhanced the contrast of cancer cell CT and MR images, further increasing its potential for clinical translation.

Jingfang Xiao et al. experimented with bismuth selenide nanoparticles (Bi_2_Se_3_s), combining them with adipose-derived mesenchymal stromal cells (adipose-derived MSCs) to create adipose-derived MSCs/Bi_2_Se_3_s. These nanoparticles were found to be more effectively enriched in lung tumors in tumor-bearing mice compared to bare Bi_2_Se_3_s, thereby amplifying the radiosensitivity of A549 cells and extending the survival time of mice (32). This study used an in-situ tumor model, with rigorous design and thorough verification, making it persuasive. **Table 3** describes semiconductor nanoparticles in the previous section.

**Table 3:** Types of semiconductor nanoparticles used in combination with IR in NSCLC.

Nanoparticles	Cells	Mechanism	Outcomes	Refenrence
TiO2	A549	Production of ROS through electron-hole pairs triggered by high Cerenkov radiation from X-rays.	Heightened radiosensitivity of A549 cells.	(30)
Gd-ZnO-NPs	SKLC-6	Inducing apoptosis and cell cycle arrest in G1 phase, downregulating DNA damage repair genes.	SER of 1.47 and 1.61 at concentrations of 10 and 20 μg/mL, respectively; increased radiosensitivity.	(31)
Bi2Se3s	A549	Enrichment in lung tumors when combined with adipose-derived MSCs.	Amplified radiosensitivity; extended survival time in tumor-bearing mice.	(32)

### 2.3 Other types of nano-radiosensitizers

Beyond metal and semiconductor nano-radiosensitizers, alloys, oxides, and various other nano-radiosensitizing agents have been explored. Yingming Sun et al. enhanced the solubility and stability of platinum-iron alloy nanoparticles by integrating cysteine to form FePt-Cys NPs. These nanoparticles, when combined with radiotherapy in A549 and H1975 cells, notably reduced VEGF and MMP2 expression, potentially contributing to their radiosensitizing effects (33). Similarly, Shijing Ma’s team utilized FePt NPs anchored on graphene oxide, successfully inhibiting the proliferation of A549, H460, and H1975 cells. This approach prompted autophagy, escalated ROS generation, and consequently increased NSCLC cell radiosensitivity (34). Both researches were well-conducted, but the former lacked a systemic toxicity examination while the latter lacked a dose escalation study.

MnO_2_, a widely used oxidant, demonstrated the capability to diminish reduced glutathione levels in both PC9 and TKI-resistant PC9GR cells. This activity ameliorated the tumor hypoxic environment and augmented radiotherapy effectiveness (35). The absence of animal experimentation was a flaw in it. In another study, MnO_2_ NPs, in combination with radiotherapy, enhanced ROS synthesis and activated the cGAS/STING pathway in A549 and H520 cells, triggering anti-tumor immune responses in mice (36). Feifei Li’s team synthesized gadolinium oxide nanoparticles that, post X-ray irradiation, spurred hydroxyl radical and ROS production in various cell lines, fostering cellular oxidative stress, autophagy, and enhancing radiotherapy efficacy (37). These two studies have explored the fields of anti-tumor immunity and autophagy respectively, which standed out as their highlights. Yingbo Li et al. developed pH-sensitive superparamagnetic iron oxide nanoclusters, which disintegrated in the tumor’s acidic milieu and, under X-ray exposure, intensified ROS production, lipid peroxidation, DNA damage, apoptosis, and iron death response, thereby improving H460 cell radiosensitivity (38). This research was also very solid, and it would be better if the long-term toxicity of the drug could be detected.

Selenium nanoparticles (SeNPs), a well-known inorganic nanomaterial, effectively augmented caspase-3 expression in A549 cells when used with radiotherapy, initiating apoptotic pathways leading to cell death (39, 40). Jingxia Tian and his colleagues discovered that SeNPs, in synergy with radiotherapy, significantly inhibited proliferation-associated proteins (CCND1, c-Myc) and invasion-related proteins (MMP2, MMP9) in A549 and H23 cells. This synergy also promoted apoptosis-related proteins, thereby curbing NSCLC cell migration and invasion and inducing apoptosis (41). Shiqing Nie’s team evaluated the effects of various selenium compounds in SPC-A1 cells, concluding that selenadiazole SeD exhibited the most pronounced radiosensitizing impact *in vitro* (42). All of these studies have investigated the mechanisms involved, but they lacked *in vivo* experiments that could be improved.

Thangirala et al. synthesized nano-diaminotetraacetic acid from tetraiodothyroacetic acid, a ligand of thyroid integrin αvβ3. Applied to a thymus-less H1299 xenograft tumor model in mice, this compound, upon external irradiation, achieved more significant tumor regression than radiotherapy alone (43). Min Hua Chen’s team designed hafnium-doped hydroxyapatite (Hf: HAp) nanoparticles that, in conjunction with radiotherapy, led to a substantial ROS accumulation in A549 cells, enhancing cellular damage (44). Matthias and colleagues developed lutetium phosphate nanoparticles doped with praseodymium cations, which emitted photons upon X-ray irradiation, causing DNA damage, cell cycle blockage, and thus amplifying radiosensitization in hypoxic A549 cells (45). Thao et al.’s design of lutetium phosphate nanoparticles doped with praseodymium and neodymium cations showed similar outcomes (46). The first two studies were relatively comprehensive, and further exploration *in situ* tumor irradiation and dose escalation was the direction for follow-up expansion. The last two studies attempted to simulate the *in vivo* tumor environment *in vitro*. However, if *in vivo* modeling could be conducted, it would be more convincing.

**Table 4** is a summary of the nanoparticles mentioned above.

**Table 4:** Types of other types of nano-radiosensitizers combined with IR in NSCLC.

Nanoparticles	Cells	Mechanism	Outcomes	Reference
FePt-Cys NPs	A549, H1975	Increased solubility and stability; reduction in VEGF and MMP2 expression.	Significant radiosensitization effect.	(33)
FePt NPs on Graphene Oxide	A549, H460, H1975	Inhibition of cell proliferation; induction of autophagy and increased ROS production.	Enhanced NSCLC cell radiosensitivity.	(34)
MnO2 NPs	PC9, PC9GR	Reduction in glutathione levels; improvement in tumor hypoxic environment.	Improved efficacy of radiotherapy.	(35)
MnO2 NPs	A549, H520	Increased ROS synthesis; activation of cGAS/STING pathway.	Triggered anti-tumor immune responses in mice.	(36)
Gadolinium Oxide NPs	A549, H1299, H1650	Induction of hydroxyl radical and ROS production; cellular oxidative stress and autophagy.	Enhanced radiotherapy efficacy.	(37)
pH-sensitive superparamagnetic iron oxide nanocluster	H460	pH-sensitive; promotes ROS production and lipid peroxidation.	Increased DNA damage, apoptosis, and iron death response; improved radiosensitivity.	(38)
SeNPs	A549	Increase in caspase-3 expression; activation of apoptotic pathways.	Induced cell death.	(39, 40)
SeNPs	A549, H23	Inhibition of proliferation and invasion-related proteins; promotion of apoptosis-related proteins.	Inhibited cell migration and invasion; induced apoptosis.	(41)
SeNPs	SPC-A1	Radiosensitizing impact.	Most significant radiosensitization effect *in vitro*.	(42)
Nano-diaminotetraacetic Acid	H1299	Significant tumor regression upon external irradiation.	More effective than radiotherapy alone.	(43)
Hafnium-doped Hydroxyapatite NPs	A549	Large accumulation of ROS.	Contributed to cellular damage.	(44)
LuPO4:Pr3+ NPs	A549	Emission of photons upon X-ray irradiation; DNA damage and cell cycle blockage.	Enhanced radiosensitization.	(45)
LuPO4: Pr3+, Nd 3+	A549	Similar to LuPO4:Pr3+ NPs.	Similar outcomes as LuPO4:Pr3+ NPs.	(46)

### 2.4 Nano-radiosensitizers loaded with drugs

#### 2.4.1 Loading FDA approved NSCLC chemotherapy drugs

Chemotherapy remains a cornerstone in the treatment of NSCLC and various other cancers. Agents such as platinum, paclitaxel, and pemetrexed have shown to augment radiotherapy’s effectiveness through diverse mechanisms. For instance, cisplatin disrupts the ATM pathway crucial for repairing cellular damage caused by irradiation (47). Paclitaxel (PTX) orchestrates NSCLC cells to pause at the radiosensitive G2/M phase (48), while pemetrexed impedes nucleotide precursor synthesis, impacting DNA repair (49). Nanotechnology’s advent has pioneered the encapsulation and delivery of these radiosensitizing chemotherapeutics directly into lung tumors, forging a novel and synergistic approach in chemotherapy-radiotherapy treatments.

PTX, known for its poor water solubility, is traditionally dissolved in polyoxyethylated castor oil, a substance linked to allergic reactions and neurotoxicity (50). In 2006, T Negishi and team leveraged NK105, a micellar nanoparticle formulation of PTX, in mice inoculated with Lewis cells. Administering this formulation followed by X-ray irradiation led to enhanced efficacy compared to conventional PTX, notably inducing a higher rate of tumor cell arrest in the G2/M phase (51). The experimental group setup in this study was quite reasonable. However, in the absence of in-vitro experiments to determine the IC50 of NK105, directly using a dose of 45 mg kg^−1^ *in vivo* might not be quite suitable. Genexol-PM, another micellar formulation of paclitaxel free from polyoxyethylated castor oil, mirrored NK105’s antitumor effects and radiosensitization properties (52). The study would be improved by incorporating a group that was solely treated with the drugs. Exploring further, Wheemoon et al. developed LOXab NPs by fusing LOX antibodies with PTX, yielding a highly targeted approach against A549 cells, resulting in increased apoptosis and radiosensitization (53). Despite the unirradiated tumor sites not presenting a remarkable abscopal effect, the study which utilized a model of tumors implanted on both sides of mice was well-designed. Additionally, the FDA has approved an albumin-bound form of paclitaxel for NSCLC’s frontline treatment, which will be described in detail in later sections.

In the realm of platinum-based treatments, platinum nanoparticles, particularly those of cisplatin-NPs and carboplatin-NPs, are garnering significant attention. Y Hao and his colleagues observed that cisplatin-NPs and carboplatin-NPs, when administered via inhalation, concentrate more effectively in lung tumors, enhancing radiotherapy’s synergistic effects while minimizing normal tissue toxicity (54). Utilizing liposomes, nanoscale drug carriers, coupled with EGFR antibodies and cisplatin, showed promising results in targeting mouse A549 xenograft tumors and boosting radiosensitivity (55). Joseph et al. crafted cisplatin precursor nanoparticles through alkali-catalyzed sol-gel polymerization and modified them with polyethylene glycol (PEG) to evade mononuclear phagocytic system uptake, resulting in superior performance *in vivo* and *in vitro* (56). Maofan Zhang et al. synthesized PEG-PLGA NPs encapsulating etoposide and cisplatin, achieving significant SERs (1.6 and 1.65) in 344SQ and H460 cells without added toxicity (57). Ling-Yu Chen and his team developed albumin-based cisplatin-gold nanoparticles (Au-cisplatin NPs), demonstrating remarkable superiority in tumor control and anti-tumor immunity when combined with radiotherapy (58). The aforementioned five studies each possessed their unique attributes, including features such as inhalation drug delivery and liposome encapsulation. Apart from the first study which did not undertake a safety evaluation, the remaining investigations enhanced efficacy without amplifying toxicity, representing particular value in terms of clinical application utility.

Gemcitabine, a first-line therapy for advanced NSCLC, saw innovation through Ji Liu et al.’s work, who attached RGDc peptides to lipid GNPs loaded with gemcitabine. Activated by near-infrared light, this combination hindered tumor growth and bolstered radiosensitivity by facilitating ROS production in NSCLC cells (59). For advanced NSCLC, doxorubicin (DOX) serves as a second-line treatment. Jing Wang et al. prepared epidermal growth factor-modified adriamycin nanoparticles (EGF@DOX-NPs), targeting cells overexpressing EGFR, significantly heightening A549 cells’ radiosensitivity both *in vitro* and *in vivo* (60). Recognizing the high expression of Glucose-regulated protein 78 on NSCLC surfaces, Abhay et al. employed Glucose-regulated protein 78 targeting peptides with DOX liposomes, enhancing drug delivery efficiency and markedly improving radiotherapy efficacy in both A549 and H460 cells (61). These three studies also boasted robust designs. Their future exploration lies in the field of in-situ tumor studies.

There is **Table 5** summarizing nano-radiosensitizers loaded with FDA approved chemotherapy drugs in NSCLC.

**Table 5:** Types of nano-radiosensitizers loading FDA approved NSCLC chemotherapy drugs combined with IR in NSCLC.

Nanoparticle	Cells	Mechanism	Outcomes	Reference
NK105	Lewis	Tumor cell arrest in the G2/M phase.	More effective than conventional PTX	(51)
Genexol-PM	A549, H460	controlled drug release.	Antitumor effect and radiosensitization	(52)
LOXab NPs	A549	High targeting; increased cell apoptosis.	Radiosensitization.	(53)
Cisplatin-NPs and carboplatin-NPs	LLC	Administered via inhalation or intravenous	Higher concentration in lung tumors by inhalation; better synergistic effect with radiotherapy.	(54)
Cisplatin-incorporated liposomes	A549	Highly targeting tumors	Improved radiosensitivity.	(55)
Cisplatin precursor NPs	A549, H460	Reduced mononuclear phagocytosis system uptake.	Outperformed other treatment groups.	(56)
PEG-PLGA NPs (Etoposide & Cisplatin)	344SQ, H460	Increases in the intensity of the apoptosis marker cleaved caspase 3.	SERs of 1.6 and 1.65 respectively; no additional toxicity.	(57)
Au-cisplatin NPs	A549, H520, Lewis	enhanced recruitment of effector tumor-infiltrating immune cells	Superior tumor control and anti-tumor immunity compared to radiotherapy alone.	(58)
Lipid GNPs (Gemcitabine)	NSCLC cells	RGDc peptide on lipid GNPs loaded with gemcitabine; activated by near-infrared light.	Inhibited tumor proliferation; enhanced radiosensitivity.	(59)
EGF@DOX-NPs	A549	Highly targeting tumors	Improved radiosensitivity *in vitro* and *in vivo*.	(60)
Glucose-regulated protein 78 targeting peptide-Dox liposomes	A549, H460	Efficient drug delivery;	Enhanced radiotherapy efficacy.	(61)

#### 2.4.2 Loading FDA approved chemotherapy drugs for other tumors

Olaparib, an FDA-approved poly (ADP-ribose) polymerase inhibitor, plays a pivotal role in inhibiting poly (ADP-ribose) polymerase, essential for repairing radiation-induced DNA damage. It’s widely recognized for its efficacy in treating ovarian, breast, pancreatic cancer and various other cancers (62–64). Min Wu and colleagues innovatively synthesized Olaparib-NPs, which demonstrated a significantly higher SER of 3.81 in A549 cells compared to free Ola’s 1.66, without introducing additional toxicity (65). Similarily, if in-situ tumor research was conducted, it could make their studies more intriguing.

#### 2.4.3 Loading natural anti-tumor compounds

Curcumin (Cum), known for its antitumor properties in lung cancer, faces challenges in clinical use due to low solubility and bioavailability. Overcoming this, Cum-NPs, made by encapsulating Cum with polyvinylpyrrolidone-polycaprolactone, significantly enhanced apoptosis in A549 cells compared to free Cum, thereby enhancing the efficacy of radiotherapy (with a SER at 10% cell survival of 1.55 versus 1.13) (66).

Cannabinoids, active compounds in cannabis, are able to inhibit tumor growth. Wilfred and his team attached cannabinoids to ‘nanoparticle drones’ using gold nanoparticles, targeting lung tumors in a transgenic mouse NSCLC model. Administered by inhalation, these drones improved radiosensitivity while minimizing side effects (67).

Maytansinoid DM1, an alkaloid with cancer-fighting properties linked to maytansine, has been further optimized by Shi Gao’s group. They nitrosylated DM1 to produce DM1-NO and then loaded it onto PLGA-βPEG nanoparticles, creating DM1-NO PLGA-NPs that targeted NSCLC effectively. X-ray irradiation breaks the drug’s S-N bond, releasing DM1 and nitric oxide (NO), which interacts with ROS to form free radicals and block cells in the G2/M phase, enhancing radiosensitizing effect on H1299 cells (68).

Baicalein, an active anticancer agent derived from Scutellaria baicalensis, suffers from low bioavailability. This challenge was addressed by formulating it into solid lipid NPs. Applied to A549 cells, solid lipid NPs increased ROS and apoptosis, sensitizing them to radiotherapy, while also providing radioprotection in normal cells (69).

Absolutely, natural anti-tumor substances extracted from plants in nature have advantages such as being inexpensive and readily available. Figuring out how to better deliver them to tumors, enhance anti-tumor effects, and reduce toxic side effects presents a significant area of research. The studies above have provided good examples.

Presented below is **Table 6**, summarizing nano-radiosensitizers that encapsulate FDA-approved chemotherapy drugs for various tumor treatments and natural anti-tumor compounds.

**Table 6:** Types of nano-radiosensitizers loading FDA approved chemotherapy drugs for other tumors or natural anti-tumor compounds in combination with IR in NSCLC.

Nanoparticles	Cells	Mechanism	Outcomes	Reference
Olaparib-NPs	A549	Inhibition of DSB repair and the promotion of cell apoptosis.	SER of 3.81, without additional toxicity.	(65)
Cum-NPs	A549	Increased apoptosis.	SER10 of 1.55.	(66)
Nanoparticle Drones	Transgenic mouse NSCLC model	Attached to GNPs, targeting lung tumors; administered by inhalation.	Improved radiosensitivity, minimized side effects.	(67)
DM1-NO PLGA-NPs	H1299	Released DM1 and NO upon X-ray irradiation, blocking cells in G2/M phase.	Enhanced radiosensitivity.	(68)
solid lipid NPs	A549	Increased ROS and apoptosis.	Sensitized cells to radiotherapy, provided radioprotection in normal cells.	(69)

#### 2.4.4 Loading drugs targeting high expression biomarkers in NSCLC

Jinghui Zhang’s team discovered that H1299 cells, which survived repeated X-ray irradiation, exhibit high expression of ALDH1 and CD133 proteins. Notably, in the ALDH1^+^ CD133^+^ NSCLC cell subset, miR-21 and miR-95 levels were significantly elevated compared to the ALDH1^-^ CD133^-^ group. Addressing this, the researchers used calcium carbonate nanoparticles to deliver anti-miR21 and anti-miR95 to NSCLC cells. This approach markedly inhibited tumor growth and enhanced radiosensitivity in H1299 cells, potentially by upregulating PTEN, SNX1, and SGPP1, while concurrently suppressing the PI3K-Akt pathway (70).

The overexpression of the MUC1-C subunit, commonly found in NSCLC tumors, led Alexandre et al. to develop MUC1-C antibody-conjugated Gd-based nanoparticles. These nanoparticles achieved a SER of 1.86 in H460 cells and showed prolonged retention in tumor models, boosting the effectiveness of fractionated radiotherapy (71).

Specificity protein 1 (SP1), often overexpressed in NSCLC, was targeted by GNPs-siSP1, comprising siSP1 and gold nanoparticles. GNPs-siSP1, easily internalized by A549 cells, reduced SP1 expression, upregulated granzyme B, and arrested cells in the G2/M phase, thereby enhancing radiosensitivity with SERs of 2.09 and 2.13 at 10 nM and 20 nM concentrations, respectively (72).

KRAS is a member of the human Ras gene family, with KRAS mutations present in 20% -25% of NSCLCs. Linlin Yang et al. engineered EGFR_apt_-3WJ-siKRAS^G12C^ nanoparticles targeting KRAS mutations, effectively reducing KRAS^G12C^ expression in H2122, H2030, and H1299 cells. This innovation inhibited the downstream MAPK pathway and amplified the tumor-suppressive impact of radiotherapy (73).

#### 2.4.5 Loading drugs targeting genes

HPNAS-4 has been recognized as a pro-apoptotic gene. When plasmids carrying the HPNAS-4 gene were delivered to NSCLC cells using liposomes, there was a notable overexpression of the hPNAS-4 protein. This led to increased apoptosis in A549 and Lewis cells, significantly enhancing the efficacy of radiotherapy (74).

In another study, Chang’s team developed plasmids that combined radiation-responsive Egr1 promoters with hypoxia-responsive enhancers. These plasmids, when introduced into A549 cells via liposomes, triggered the overexpression of the pro-apoptotic protein Smac. This intervention promoted apoptosis and caused G2/M phase arrest in the cells, ultimately improving the radiosensitivity of A549 cells under hypoxic conditions (75).

Nowadays, people can initially select the research targets by screening the genes that are differentially expressed in normal tissues and tumor tissues from the public database. How to further screen the preliminary data to pinpoint the specific molecules and design rigorous experiments around them is a topic worthy of deep investigation.

**Table 7** presents an overview of nano-radiosensitizers encapsulating drugs specifically aimed at targeting highly expressed biomarkers or genes in NSCLC.

**Table 7:** Types of nano-radiosensitizers loading drugs targeting high expression biomarkers or genes in NSCLC in combination with IR.

Nanoparticles	Cells	Mechanism	Outcomes	Reference
Calcium Carbonate Nanoparticles	H1299	Delivery of anti-miR21 and anti-miR95; upregulation of PTEN, SNX1, SGPP1.	Inhibited tumor growth; enhanced radiosensitivity.	(70)
MUC1-C Antibody-Conjugated Gd-based NPs	H460	Prolonged retention in tumors.	SER of 1.86.	(71)
GNPs-siSP1	A549	Reduced SP1 expression; upregulated granzyme B; G2/M phase arrest.	SERs of 2.09 and 2.13.	(72)
EGFRapt-3WJ-siKRASG12C NPs	H2122, H2030, H1299	Targeting KRAS mutations; inhibition of MAPK pathway.	Reduced KRASG12C expression; amplified radiotherapy impact.	(73)
HPNAS-4 Gene Plasmids in Liposomes	A549, Lewis	Overexpression of hPNAS-4 protein; induced apoptosis.	Increased apoptosis; enhanced radiotherapy efficacy.	(74)
Egr1- hypoxia-responsive enhancers Plasmids in Liposomes	A549	Overexpression of pro-apoptotic protein Smac; G2/M phase arrest.	Promoted apoptosis; improved radiosensitivity under hypoxic conditions.	(75)

#### 2.4.6 Loading multiple drugs

The exceptional targeting and loading capacity of nanoparticles allow for the efficient delivery of increased drug quantities to tumors. Jyothi et al. developed multifunctional dual drug loaded nanoparticles, encapsulating the DNA-PK inhibitor NU7441 and gemcitabine in superparamagnetic iron oxide nanoparticles, and augmented them with folate for targeting folate receptors, which are overexpressed in various cancers, including NSCLC. These folate-coupled MDNPs demonstrated prolonged retention in NSCLC compared to their uncoupled counterparts. Upon reaching tumors, multifunctional dual drug loaded nanoparticles underwent vesicle-mediated endocytosis, releasing their contents in the acidic environment of endosomes or lysosomes, thereby exerting cytotoxic effects and enhancing the radiosensitivity of A549 and H460 cells (76). Roshni and his colleagues co-loaded NU7441 and cisplatin into nanoparticles, attaching them to antibodies targeting the Ephrin receptor A2, prevalent in NSCLC. These Ephrin-coupled NPs significantly increased A549 cells’ radiosensitivity (77). Kin et al. crafted diblock copolymer nanoparticles for the sequential release of warfarin and docetaxel into H460 cells, which, when combined with radiotherapy, outperformed other treatment modalities (78).

Moataz’s team developed C-siPLK1-NPs carrying cetuximab, an EGFR-targeting monoclonal antibody, and siPLK1, an siRNA targeting the mitotic regulator PLK1. These nanoparticles effectively targeted A549 and H460 cells, reduced PLK1 expression, induced G2/M blockade, and acted as radiosensitizers (79). Shuzhen Chen et al. utilized Fe_3_O_4_ magnetic NPs to carry SiBIRC5 and BIRC5 antisense oligodeoxynucleotides, addressing the upregulated anti-apoptotic protein BIRC5 in NSCLC. These magnetic NPs enhanced drug uptake in A549 and H460 cells, diminished BIRC5 expression, and increased death receptor 5 expression, thereby improving radiotherapy’s therapeutic effect. Moreover, Magnetic field guidance further amplified drug enrichment in tumors (80). Jinghua Han’s team engineered nanoparticles loaded with DOX and 5-aminolevulinic acid, a radiosensitizer, coupled with a neurotensin receptor 1 ligand to target neurotensin receptor 1-high-expressing H1299 cells. The acidic tumor environment triggered DOX release for cytotoxic impact, while 5-aminolevulinic acid targeted mitochondria, enhancing radiation-induced oxidative stress and the overall efficacy of radiation therapy in treating H1299 cells (81).

Packaging two types of drugs into nanoparticles, one being an FDA-approved chemotherapy drug and the other a radiosensitizer, has become the choice of many researchers. On the basis of guaranteeing precision in tumor targeting and releasing medications in the optimal order, this approach is capable of amalgamating traditional drugs, consequently contributing to a more efficacious anti-cancer impact.

#### 2.4.7 Loading other types of drugs

XIAP, an inhibitor of apoptotic protease-3, plays a crucial role in cellular apoptosis. By using liposomes to transport siXIAP into both p53 wild-type and mutant H1299 cells, researchers significantly enhanced radiosensitivity, particularly in p53-mutant cells (82). This study also lacked *in vivo* experiments.

Survivin, known as a radioresistance factor, can be counteracted by mS-T34A, a plasmid that prevents survivin from binding to activated caspase-9. Qing-Zhong’s team used liposomes to create Lip-mS, effectively increasing cell apoptosis, inhibiting tumor angiogenesis, and enhancing radiosensitivity in Lewis cells (83).

The DNA double-strand repair inhibitor KU55933, known for inhibiting DNA double-strand break repair in H460 cells, saw improved radiosensitizing effects when loaded onto nano-lipid polymers by Xi Tian and his colleagues (84).

Radiotherapy of primary tumors in concert with immunoadjuvants can lead to regression of tumors out of the radiation field, which is called as abscopal effect (85). Yao Hao et al. used biodegradable nanopolymers encapsulated with anti-CD40 antibody to significantly enhance radiotherapy’s effect in Lewis cells, slowing tumor growth both within and outside irradiated fields and improving mice survival (86).

All three studies mentioned above were deficient in drug safety evaluations. There was room for enhancement in this area.

Lonidamine, an oxidative phosphorylation inhibitor, was innovatively combined with mitochondria-targeted triphenylphosphine cation and encapsulated in liposomes by Saijun Wang’s team to form TPP- Lonidamine@Lip, which activated AMP-dependent protein kinase through oxidative phosphorylation inhibition, reduced PD-L1 expression, and bolstered anti-tumor immunity. Additionally, it reversed tumor hypoxia, making A549 cells more radiosensitive (87). While this research was quite comprehensive, it would be more beneficial if an in-situ tumor model was utilized.

Presented below, **Table 8** enumerates various nano-radiosensitizers designed to carry other types of drugs or multiple drugs.

**Table 8:** Types of nano-radiosensitizers mutiple drugs in combination with IR in NSCLC.

Nanoparticles	Cells	Mechanism	Outcomes	Reference
Folate-coupled multifunctional dual drug loaded NPs encapsulating NU7441 and gemcitabine	A549, H460	Endocytosis and release in acidic environment.	Increased apoptosis; enhanced radiosensitivity.	(76)
Ephrin-coupled NPs	A549	Co-loaded with NU7441 and cisplatin; targeting NSCLC.	Significantly increased radiosensitivity.	(77)
Diblock Copolymer NPs	H460	Sequential release of warfarin and docetaxel.	Superior therapeutic effect compared to other treatments.	(78)
C-siPLK1-NPs carrying Cetuximab and siPLK1	A549, H460	Reduced PLK1 expression; induced G2/M blockade.	Acted as radiosensitizer.	(79)
Fe3O4 magnetic nanoparticles loaded with SiBIRC5 and BIRC5 antisense sequence	A549, H460	Reduced BIRC5 expression; increased death receptor 5 expression.	Improved radiotherapy efficacy.	(80)
NTSR1 ligand-coupled NPs loading DOX & 5-aminolevulinic acid	H1299	Targeted cytotoxic effects; amplified radiation-induced stress.	Enhanced killing effect of radiation.	(81)
siXIAP Liposomes	H1299	Transporting siXIAP into p53 wild-type/mutant cells.	Significantly enhanced radiosensitivity, especially in p53-mutant cells.	(82)
Lip-mS combining mS-T34A plasmid with liposomes	Lewis	Inhibit survivin.	Increased apoptosis, inhibited tumor angiogenesis, enhanced radiosensitivity.	(83)
Nano-lipid Polymers with KU55933	H460	Inhibit DNA repair.	Improved radiosensitizing effect.	(84)
Anti-CD40 Nanopolymers	Lewis	Encapsulated with anti-CD40 antibody for radio-immunotherapy.	Slowed tumor growth in and out of irradiated fields, improved survival.	(86)
TPP- Lonidamine@Lip	A549	Activated AMPK, reduced PD-L1.	Enhanced anti-tumor immunity, increased radiosensitivity.	(87)

### 2.5 Combination of nano-radiosensitizers with radiotherapy plus phototherapy, thermotherapy, or immunotherapy

#### 2.5.1 Nano-radiosensitizers+ Radiotherapy+ Phototherapy

Wensha Yang and his colleagues utilized polyethylene glycol-coated, amine-functionalized semiconductor nanocrystals (QDs), which, under X-ray irradiation, excited photons to activate photosensitizers. Applied to H460 cells, these QD-photosensitizer conjugates, in conjunction with radiotherapy, were more effective in cell destruction compared to other treatments (88).

Jun Ma’s team developed Ce6/PTX 2-NP/G@NHs, polymer nanoparticles composed of the paclitaxel prodrug and photosensitizer Ce6. These nanoparticles were readily uptaken by NSCLC cells, with Ce6 promoting ROS production and creating a hypoxic environment under laser irradiation. This induced PTX release, directly killing cancer cells and inactivating the PI3K/AKT pathway. As a result, the nanoparticles increased apoptosis in A549 cells, especially when combined with radiotherapy (89).

Cypate (Cyp), an indocyanine green derivative, generates heat under near-infrared light irradiation. Cyp-polymethylmethacrylic acid-Fe@MSCs, comprising polymethylmethacrylic acid nanoparticles loaded with iron and Cyp, encapsulated in mesenchymal stem cell membranes, were more effective in targeting Lewis cells, leading to significant tumor shrinkage under laser and X-ray irradiation (90).

#### 2.5.2 Nano-radiosensitizers+ Radiotherapy+ Thermotherapy

Magnetic nanoparticle clusters (MNCs), wrapped with polyacrylic acid for biocompatibility, generated heat in an alternating magnetic field and were used for cancer thermotherapy. Jia Ma et al. found that MNCs-treated H460 cells showed increased expression of Hsp70 and caspase-3 under alternating magnetic field and radiotherapy, significantly suppressing tumors more than other treatments (91).

Mn-Zn ferrite magnetic nanoparticles, similar to MNCs, were integrated into PEG-β-PCL block copolymer micelles and modified with hyaluronic acid targeting A549 cells. In tumors, Mn-Zn ferrite magnetic NPs not only generated heat, but also raised oxygenation levels under alternating magnetic field, thereby enhancing A549 cells’ radiosensitivity (92).

Tsl-MTX, comprising 1-methylxanthine and temperature-sensitive liposomes, released its contents upon local heating of tumors, achieving pronounced tumor regression, particularly when combined with radiotherapy (93).

#### 2.5.3 Nano-radiosensitizers+ Radiotherapy+ Immunotherapy

Yun Hu’s team explored the potential of hafnium oxide nanoparticles NBTXR3 in an anti-PD1 resistant lung cancer model 344SQR. Only the group receiving NBTXR3 with high and low-dose irradiation (12Gy*3F and 1Gy*2F) plus immunotherapy (anti-PD1 and anti-CTLA-4) exhibited significant CD8^+^ T cell/Treg cell ratio improvement and tumor regression, highlighting NBTXR3’s synergy with radiation and immunotherapy (94). In further studies, combining NBTXR3 with radiotherapy and inhibitors of TIGIT and LAG3, the team demonstrated a significantly enhanced treatment effect, supporting the clinical translation of NBTXR3 (95).

Ying Wang et al. found that cisplatin-loaded nanoparticles induced CXCL10 secretion in tumors. Post-irradiation, there was increased CD8^+^ T cell infiltration in both irradiated and unirradiated tumors. Combining this with anti-PD1 therapy resulted in significantly greater tumor regression, illustrating cisplatin-loaded NPs’ role in boosting anti-tumor immunity post-radiotherapy and achieving an abscopal effect (96).

Leveraging nanotechnology to enhance radiation sensitization, and pairing it with phototherapy or thermotherapy could significantly improve the tumor-targeting ability and anti-cancer efficacy of drugs. Moreover, immunotherapy has emerged as one of the most promising research fields in oncology in recent years, suggesting that boosting anti-cancer immunity holds substantial potential for exploration. By integrating the use of nanomedicine, it allows us to combine differing methods of cancer treatment, thus offering profound clinical implications.

Presented next is **Table 9**, showcasing nano-radiosensitizers that are employed in combination with radiotherapy plus phototherapy, thermotherapy, or immunotherapy.

**Table 9:** Types of nano-radiosensitizers in combination with IR plus phototherapy, thermotherapy, or immunotherapy.

Nanoparticles	Combined teatment	Cells	Mechanism	Outcomes	Reference
QDs-Photosensitizer Conjugates	Phototherapy	H460	QDs, upon irradiation, excited photons to activate photosensitizers.	Enhanced cell destruction.	(88)
Ce6/PTX 2-NP/G@NHs	Phototherapy	A549	Ce6 promoted ROS production and created a hypoxic environment under laser. Inactivating the PI3K/AKT pathway	Increased apoptosis.	(89)
Cyp-polymethylmethacrylic acid-Fe@MSCs	Phototherapy	Lewis	Cypate generated heat under near-infrared light; targeting tumors.	Significant tumor shrinkage.	(90)
PAA modified MNCs	Thermotherapy	H460	Heat generation in an alternating magnetic field; increased expression of Hsp70 and caspase-3.	Significant tumor suppression.	(91)
HA modified magnetic NPs	Thermotherapy	A549	Heat generation and increased oxygenation in tumors; modified with hyaluronic acid.	Enhanced radiosensitivity of tumors.	(92)
Tsl-MTX	Thermotherapy	A549	Precise content release upon local heating.	Pronounced tumor regression.	(93)
NBTXR3	Immunotherapy	344SQR	CD8+ T cell/Treg cell ratio improvement.	Tumor regression; abscopal effect	(94, 95)
CDDP-NPs	Immunotherapy	Lewis	CXCL10 secretion in tumors; increased CD8+ T cell infiltration	Significant tumor regression; abscopal effect.	(96)

## 3 Clinical studies of nanomaterials involved in radiotherapy in NSCLC

The combination of paclitaxel and platinum represents a primary treatment option for advanced NSCLC (97). Paclitaxel’s poor water solubility often necessitates its dissolution in lox, which is linked to allergic reactions and neurotoxicity (50). In a significant development, Neil Desai et al., in 2006, synthesized 130nm albumin-paclitaxel particles (nab-P), which demonstrated enhanced anti-tumor effects and reduced toxicity compared to traditional paclitaxel (98). The FDA approved nab-P for first-line NSCLC treatment in 2012.

The inaugural phase I clinical study combining nab-P and radiotherapy in NSCLC was conducted by V. L. Keedy’s team in 2010. Administering a 66Gy/33F + nab-P+ carboplatin regimen to 11 pts with locally advanced NSCLC, they observed 9 partial responses (PR), 1 stable disease (SD), and 1 withdrawal post-consent. The most severe adverse event was grade 3, indicating that a 40mg/m^2^ weekly nab-P regimen is safer in combination with carboplatin and radiotherapy (99).

In 2017, Kan Wu et al. reported a phase II clinical trial of radiotherapy combined with carboplatin + 60mg/m^2^ nab-P in locally advanced squamous cell lung cancer. Of 8 pts, 5 showed PR, 2 had SD, and 1 experienced progressed disease (PD). The median progression-free survival (mPFS) and overall survival (mOS) were 12.1 and 15.2 months, respectively (100).

These two studies represented the earliest phase I or II clinical trials involving the use of nab-P l during radiotherapy for NSCLC pts, with the disadvantage of having few participants. In subsequent clinical trials, the number of participants was relatively increased, compensating for this drawback.

Ryo Shimoyama et al. initiated a phase 3 clinical trial in 2020, investigating synchronized carboplatin with or without nab-P during radiotherapy for stage III NSCLC. Results of this ongoing study are highly anticipated (101).

Up to now, clinical studies on nab-P plus carboplatin during radiotherapy are limited. Although the combination is a class I recommendation for NSCLC patients, conventional paclitaxel plus carboplatin remains the recommended synchronous chemotherapy regimen during radiotherapy. Further research is needed to validate nab-P plus carboplatin as synchronized chemotherapy in NSCLC.

In parallel, C. Shen et al. conducted a multicenter, open-label phase I study on NBTXR3, involving patients with various cancers, including lung cancer. Early results from this ongoing trial, which is still recruiting patients, have shown overall tumor regression in 8 out of 9 patients, including 4 with lung cancer. These promising findings highlight the potential of combining SBRT with NBTXR3 and anti PD-1 therapy in solid tumors, and emphasize the necessity for further research involving more patients with inoperable NSCLC and other malignancies (102).

More clinical trials are described in **Table 10** (99–108).

**Table 10:** Clinical studies of nanomaterials involved in radiotherapy in NSCLC.

Nanoparticles	Phase	Pts	Cancer	Regimen	Outcomes	Reference
nab-P	I	11	Locally Advanced NSCLC	66Gy/33F + 40mg/m^2^ nab-P + carboplatin.	9 PRs, 1 SD, 1 withdrawal; most severe adverse event was grade 3.	(99)
nab-P	II	8	Locally Advanced Squamous Cell Lung Cancer	66Gy/33F + 60mg/m^2^ nab-P + carboplatin	ORR 75%; mPFS 12.1 months; mOS 15.2 months.	(100)
nab-P	III	Recruiting	Stage III NSCLC	60Gy/30F+ carboplatin ± 30 mg/m^2^nab-P	ongoing	(101)
nab-P	I/II	58	Locally Advanced NSCLC	60Gy/30F+ weekly nab-P (40 or 50 mg/m^2^) + carboplatin	Feasible with weekly nab-P at 50 mg/m^2^; mPFS 11.8 months; 2-year OS 66.1%. ORR 76.8%	(103)
nab-P	I	14	Locally Advanced NSCLC	60Gy/30F+ weekly nab-P (40, 60 or 80 mg/m^2^) + carboplatin	ORR 71.4%; Recommendation for nab-P at 40 mg/m^2^	(104)
nab-P	I	19	Locally Advanced NSCLC	64Gy/32F+ weekly nab-P (30 or 40 mg/m^2^) + carboplatin	Recommendation for weekly nab-P at 40 mg/m^2^; mPFS 13.4 months; ORR 76.5%	(105)
nab-P	I/II	28	Locally Advanced NSCLC	60Gy/30F+ biweekly nab-P (100 mg/m^2^) + carboplatin	ORR 96.4%; mPFS 18.2 months; 2-year OS 67.8%	(106)
nab-P	I	18	stage III NSCLC	60Gy/30F+ biweekly nab-P (60, 80 or 100 mg/m^2^) + carboplatin	Recommendation for biweekly nab-P at 100 mg/m^2^	(107)
nab-P	II	10	Locally Advanced NSCLC	60Gy/30F+ weekly nab-P (40 mg/m^2^) + carboplatin	ORR 40%; mPFS 6.7 months;	(108)
NBTXR3	I	Recruiting	Various Cancers including Lung Cancer	SBRT + NBTXR3 + anti PD-1 therapy.	Overall tumor regression in 8 out of 9 pts; ongoing trial.	(102)

## 4 Conclusions

The burgeoning advancement of novel nanomaterials in biomedicine offers an array of possibilities for augmenting the efficacy of radiotherapy in treating tumors clinically. This article delves into various nano-radiosensitizers, which either intrinsically heighten the radiosensitivity of NSCLC cells or act as carriers for radiosensitive drugs, thereby localizing their delivery to tumors. This synchronization of radiotherapy and targeted drug action amplifies the radiosensitivity of NSCLC cells through diverse mechanisms.

Nonetheless, the clinical adoption of these nano-radiosensitizers is not without hurdles. Unlike traditional drugs, nanodrugs, due to their smaller sizes, may not biodegrade, potentially leading to long-term accumulation in the body and resultant unknown toxic side effects, which limits their utility in radiotherapy. As such, the future direction in this field lies in bolstering biocompatibility, enhancing tumor-targeting capabilities, optimizing drug loading capacity, and cutting costs without compromising biosafety. Moreover, the multifunctionality of nanoparticles should be fully harnessed to facilitate tumor imaging and to extend the amalgamation of various treatments such as radiotherapy, chemotherapy, thermotherapy, phototherapy, and immunotherapy, thereby transcending the restrictions of singular treatment modes. Current clinical trials predominantly involve small sample sizes and have follow-up periods generally ranging from one to two years. Hence, there is a pressing need for more comprehensive studies with extended follow-up periods to evaluate the long-term efficacy of nano-radiosensitizers.

In tandem with this, the merging of radiosensitizers with proton therapy is viewed as a promising avenue of future development. Proton therapy, in contrast to traditional radiotherapy, offers specific dosimetric advantages and fewer off-target effects. Therefore, coupling proton therapy with nano-radiosensitizers corresponds more closely with the objectives of precision medicine. Nonetheless, up to this point, scant researches have been conducted on the combination of radiosensitizers with proton therapy in the treatment of NSCLC.

Researchers such as Bronk treated lung cancer cells by nanoscaffold, discovering it amplified the effectiveness of radiotherapy (109). In addition, Yun’s team inoculated one side of a mouse limb with PD-1 inhibitor-resistant 344SQR cells and treated them with NBTXR3, PD-1 inhibitors, or proton therapy. After 76 days, they inoculated 344SQR cells on the other side again. As a result, they found that mice subjected to the triple combination therapy displayed increased infiltration and activation of cytotoxic immune cells, underscoring remarkable anti-tumor treatment effects (110). While neither study compared proton therapy with X-ray therapy, the outstanding efficacy of proton therapy should not be sidelined. The future holds the promise of extensive research exploring the amalgamation of nano-radiosensitizers with proton therapy.

Despite these hurdles, the field of nano-radiosensitizers teems with immense potential. As studies advance, it is entirely conceivable that nano-radiosensitizers could emerge as a favored option in the treatment arsenal for NSCLC, thereby ushering in novel dimensions in cancer therapy.

## Author contributions

XH: Conceptualization, Methodology, Project administration, Visualization, Writing – original draft, Writing – review & editing. JH: Data curation, Formal Analysis, Methodology, Writing – review & editing. YP: Formal Analysis, Project administration, Software, Writing – review & editing. MW: Investigation, Software, Writing – review & editing. WZ: Supervision, Validation, Writing – review & editing. XX: Supervision, Validation, Writing – review & editing. CZ: Funding acquisition, Resources, Visualization, Writing – review & editing. XW: Project administration, Resources, Visualization, Writing – review & editing. XS: Conceptualization, Writing – review & editing.
